# *In vivo* function of the murid herpesvirus-4 ribonucleotide reductase small subunit

**DOI:** 10.1099/vir.0.031542-0

**Published:** 2011-07

**Authors:** Ricardo Milho, Michael B. Gill, Janet S. May, Susanna Colaco, Philip G. Stevenson

**Affiliations:** Division of Virology, Department of Pathology, University of Cambridge, Cambridge CB2 1QP, UK

## Abstract

The difficulty of eliminating herpesvirus carriage makes host entry a key target for infection control. However, its viral requirements are poorly defined. Murid herpesvirus-4 (MuHV-4) can potentially provide insights into gammaherpesvirus host entry. Upper respiratory tract infection requires the MuHV-4 thymidine kinase (TK) and ribonucleotide reductase large subunit (RNR-L), suggesting a need for increased nucleotide production. However, both TK and RNR-L are likely to be multifunctional. We therefore tested further the importance of nucleotide production by disrupting the MuHV-4 ribonucleotide reductase small subunit (RNR-S). This caused a similar attenuation to RNR-L disruption: despite reduced intra-host spread, invasive inoculations still established infection, whereas a non-invasive upper respiratory tract inoculation did so only at high dose. Histological analysis showed that RNR-S^−^, RNR-L^−^ and TK^−^ viruses all infected cells in the olfactory neuroepithelium but unlike wild-type virus then failed to spread. Thus captured host nucleotide metabolism enzymes, up to now defined mainly as important for alphaherpesvirus reactivation in neurons, also have a key role in gammaherpesvirus host entry. This seemed to reflect a requirement for lytic replication to occur in a terminally differentiated cell before a viable pool of latent genomes could be established.

## Introduction

Herpesviruses have captured mutiple enzymes of host nucleoside metabolism. Examples include thymidine kinase (TK), which catalyses a rate-limiting step in dTTP synthesis, and ribonucleotide reductase (RNR), which makes dATP, dGTP and dCTP from the corresponding ribonucleotides (Jordan & Reichard, 1998). Like the cellular RNR, viral RNRs have a large catalytic subunit (RNR-L) and a smaller one that supplies free radicals (RNR-S). Both are essential for RNR activity (Ator *et al.*, 1986; Conner *et al.*, 1994). The relative ease of administering nucleoside analogues makes these enzymes prime targets for antiviral chemotherapy (Cameron *et al.*, 1988; Wnuk & Robins, 2006). However, effective therapy also requires that virus mutants lacking the target enzyme are avirulent. Thus, while the ubiquity of captured enzymes in alpha- and gamma-herpesvirus genomes implies one or more important roles in host colonization, it is important to understand more precisely what these roles might be.

Alphaherpesviruses require TK (Efstathiou *et al.*, 1989; Coen *et al.*, 1989) and RNR (Jacobson *et al.*, 1989; Yamada *et al.*, 1991) to reactivate from latency in neurons. The consequent avirulence of TK^−^ mutants allows acyclovir to be a potent therapy against herpes simplex virus (HSV) (Darby, 1993). Gammaherpesviruses by contrast are latent in replication-competent lymphocytes, and appear not to require TK or RNR for reactivation (Coleman *et al.*, 2003; Gill *et al.*, 2010). Gammaherpesviruses also rarely present before a self-renewing pool of infected lymphocytes is established (Hoagland, 1964). Thus, acyclovir treatment has had little effect on Epstein–Barr virus (EBV) latent loads (Yao *et al.*, 1989; Hoshino *et al.*, 2009). However, lytic replication may play a greater role in maintaining latent loads and driving disease for rhadinoviruses (gamma-2-herpesviruses) such as the Kaposi's sarcoma-associated herpesvirus (KSHV) (Ganem, 2006). Since the narrow species tropism of KSHV largely precludes *in vivo* studies, the related murid herpesvirus-4 (MuHV-4, archetypal strain MHV-68) (Stevenson *et al.*, 2009) provides a useful way to analyse conserved rhadinovirus gene functions. TK^−^ and RNR-L^−^ MuHV-4 mutants establish latency after intraperitoneal inoculation, but fail to establish significant infections via the more likely natural entry route of the upper respiratory tract (Gill *et al.*, 2009, 2010). Specifically, they show no luciferase signals in the upper respiratory tract after intranasal inoculation, and if inoculation is confined to the upper respiratory tract then infection remains detectable by PCR of spleen cells for viral DNA and serum ELISA for virus-specific IgG. Therefore, it seems that MuHV-4 must normally replicate in a terminally differentiated cell to reach its latent reservoir.

This conclusion has important implications for KSHV and EBV: despite salivary transmission being well established (Faulkner *et al.*, 2000; Pica & Volpi, 2007), how new hosts are actually infected remains unknown. However, captured host genes can evolve new functions. Gammaherpesvirus TKs are weak nucleoside kinases with large N-terminal extensions that appear to be redundant for TK activity (Gustafson *et al.*, 1998), and the KSHV TK drives cell rounding and detachment by an unknown mechanism (Gill *et al.*, 2005). Among other examples, the gammaherpesvirus ORF11 is a captured, non-functional dUTPase (Davison & Stow, 2005), and MuHV-4 FGARAT homologues have evolved important functions independent of detectable FGARAT activity (Gaspar *et al.*, 2008). Many nucleoside metabolism enzymes captured by cytomegaloviruses have lost catalytic activity, including the murine cytomegalovirus RNR-L, which has become an inhibitor of inflammation (Brune *et al.*, 2001; Upton *et al.*, 2010). Like human cytomegalovirus, murine cytomegalovirus also appears to have lost its homologues of TK and RNR-S (Rawlinson *et al.*, 1996). The HSV-1 RNR-L inhibits apoptosis (Langelier *et al.*, 2002) even though it is still part of a functional RNR. Therefore, MuHV-4 host entry via the upper respiratory tract could require TK and RNR-L functions unrelated to viral DNA replication. To test further whether viral nucleoside metabolism enzymes are required for this key aspect of the viral life cycle, we analysed the phenotype of MuHV-4 lacking RNR-S.

Cellular RNR activity is thought to be limited mainly by the availability of RNR-S (Jordan & Reichard, 1998). Non-dividing cells can produce an alternative RNR-S (p53R2) with possible roles in mitochondrial DNA replication and DNA repair, but quiescent RNR activity is only 2–3 % of that in S phase. Whether the cellular RNR-L, RNR-S or RNR-p53R2 can substitute for the corresponding viral subunits is unclear. *In vitro* screens of random MuHV-4 mutants found an RNR-S mutant to be either more attenuated than an RNR-L mutant (Song *et al.*, 2005) or equally attenuated (Moorman *et al.*, 2004). Here, we show that the MuHV-4 RNR-L has a striking perinuclear distribution independent of RNR-S, suggesting additional functions, but that MuHV-4 lacking RNR-S has a similar phenotype to that lacking RNR-L, being unable to establish a significant infection via the upper respiratory tract except at high dose. Histological examination showed individual infected cells in the olfactory neuroepithelium without RNR-S, RNR-L or TK, but no subsequent viral spread. Therefore, it appeared that MuHV-4 cannot move beyond its primary target of the olfactory neuroepithelium without supplementing cellular deoxyribonucleotide synthesis.

## Results

### Generation of MuHV-4 lacking RNR-S

**Fig. 1.**
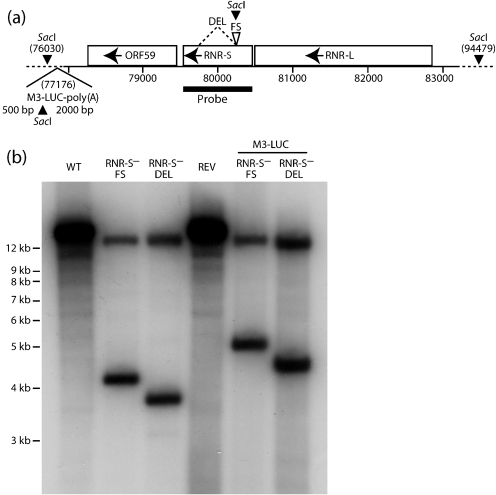
Genomic structure of MuHV-4 RNR-S mutants. (a) Schematic diagram shows the RNR-S locus, between RNR-L (ORF61) and ORF59, and the introduced DEL and FS mutations. Each introduced a *Sac*I restriction site. Also shown is the luciferase expression cassette at genomic co-ordinate 77176, which also introduces a *Sac*I site. (b) Southern blot of WT, RNR-S mutant (FS, DEL) and FS revertant (REV) viral DNA, digested with *Sac*I and probed for the RNR-S locus as shown in (a). A 18449 bp band in the WT and revertant is digested to 14215+4234 bp in the RNR-S^−^FS mutant and to 14215+3764 bp in the RNR-S^−^DEL mutant. On the M3-LUC background the predicted bands are 14215+5088 bp for RNR-S^−^FS and 14215+4618 bp for RNR-S^−^DEL.

We generated two RNR-S mutants. In each, ORF60 was disrupted after amino acid residue 72. One mutant had a frameshift at this site (RNR-S^−^FS); the other had an additional deletion of the downstream 471 bp (RNR-S^−^DEL). Each was made on both a wild-type (WT) and a luciferase-expressing virus background. We also generated a revertant of the RNR-S^−^FS mutation. A Southern blot of viral DNA (Fig. 1) confirmed that each mutant contained the expected genomic change.

### Analysis of RNR subunit expression

**Fig. 2.**
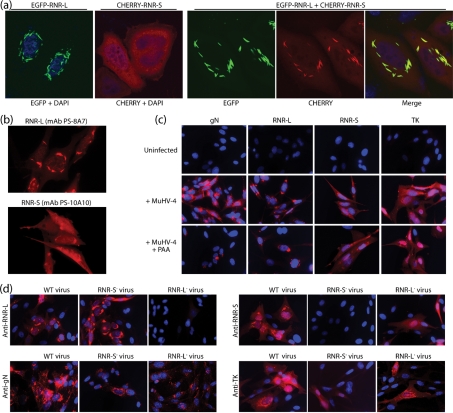
Identification of the RNR-S and RNR-L gene products. (a) HeLa cells were transfected with tagged versions of RNR-S and RNR-L, either singly or in combination. Fluorescence was examined 24 h later. RNR-L, green; RNR-S, red; co-localization, yellow. Nuclei were counterstained with DAPI (blue). The cells shown are each representative of >100 examined. BHK-21 cells were infected with WT MuHV-4 (1 p.f.u. per cell, 18 h), then fixed, permeabilized and stained for RNR-L or RNR-S with mAbs as indicated. (c) BHK-21 cells were left uninfected or infected with WT MuHV-4 (0.5 eGFP U per cell, 18 h) with or without 100 µg PAA ml^−1^ to block viral late gene expression. The cells were then fixed, permeabilized and stained for gN with mAb 3F7, for RNR-L with mAb BZ-5B2, for RNR-S with mAb PS-10A10 and for TK with mAb CS-4A5 (red). Nuclei were counterstained with DAPI (blue). (d) BHK-21 cells were infected with WT, RNR-L^−^STOP or RNR-S^−^FS MuHV-4 (eGFP U per cell, 18 h) then stained for gN, RNR-L, RNR-S or TK as in (c) to show the effect on RNR subunit disruptions on the distribution of the remaining subunit. Equivalent results were obtained with independently derived RNR-L and RNR-S mutants.

The cellular RNR subunits have a diffuse cytoplasmic distribution, based on the transfection of tagged expression constructs (Pontarin *et al.*, 2008). We transfected tagged forms of the MuHV-4 RNR-S and RNR-L to establish their distribution in a comparable manner (Fig. 2a). RNR-L alone localized to linear structures around the nucleus. RNR-S alone was diffusely cytoplasmic. In co-transfected cells, RNR-L and RNR-S co-localized in the linear structures decorated by RNR-L. Therefore, RNR-L directed RNR-S to structures not obviously associated with cellular RNR expression. RNR-L and RNR-S also adopted perinuclear localizations in MuHV-4-infected cells, with some additional diffuse cytoplasmic staining of RNR-S (Fig. 2b). Phosphonoacetic acid (PAA) treatment of the infected cells established that both RNR-S and RNR-L were early gene products, like TK but unlike glycoprotein N (Fig. 2c).

Analysis of RNR-S and RNR-L mutants (Fig. 2d) confirmed that each lacked the relevant viral gene product. The RNR-L of RNR-S^−^ MuHV-4 remained perinuclear, while the RNR-S of RNR-L^−^ MuHV-4 lost its perinuclear localization to become just diffusely cytoplasmic. Therefore, the distributions of RNR-L and RNR-S in infected cells were consistent with those observed in transfected cells.

### RNR-S^−^ virus replication *in vitro*

**Fig. 3.**
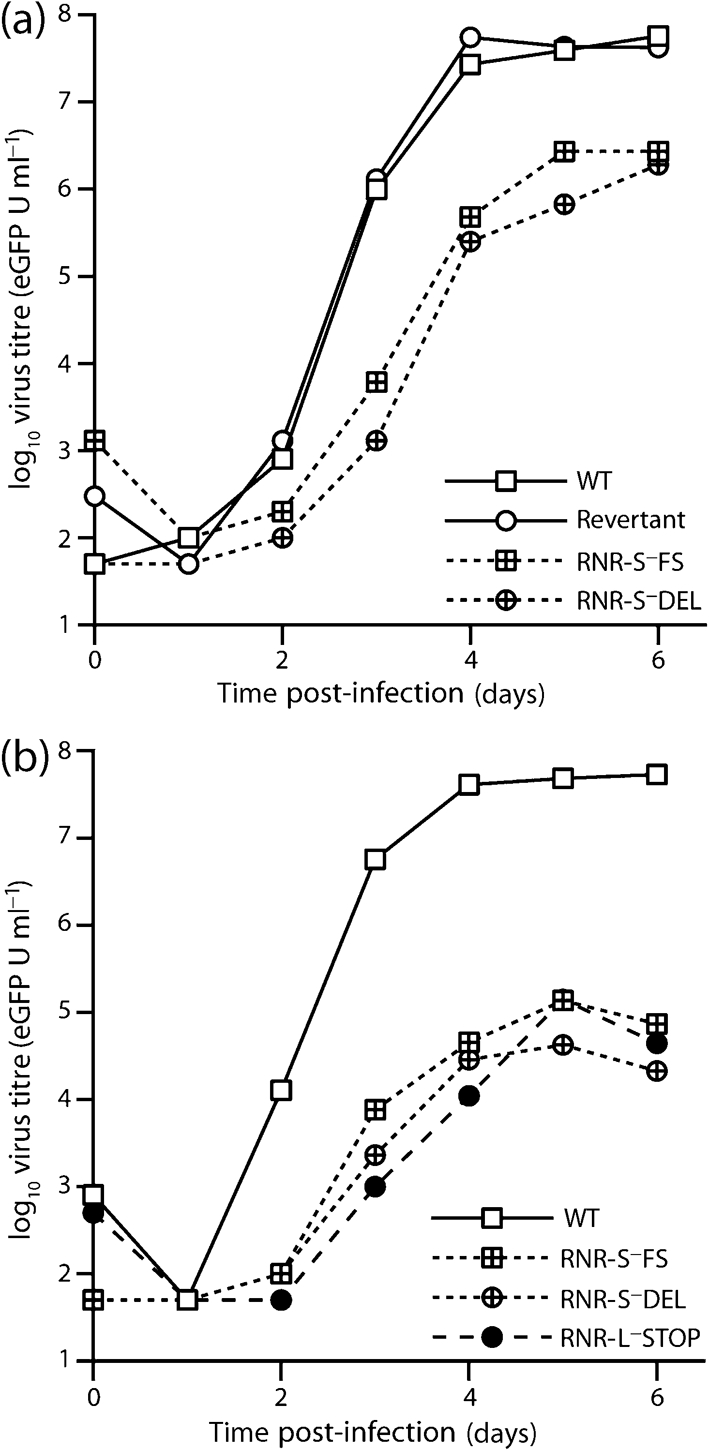
Growth of RNR-S^−^ MuHV-4 *in vitro*. (a) BHK-21 cells were infected with eGFP^+^RNR-S^+^ (WT, revertant) or eGFP^+^RNR-S^−^ (DEL, FS) viruses (0.01 eGFP U per cell) and then cultured at 37 °C. Replicate cultures were harvested every 24 h and assayed for infectivity by overnight infection of fresh BHK-21 cells and then assay of those cells for viral eGFP expression by flow cytometry. (b) BHK-21 cells were infected (0.01 eGFP U per cell) with eGFP^+^ WT, RNR-S^−^ or RNR-L^−^ (RNR-L^−^STOP) viruses. Cultures were then assayed for virus replication by eGFP infectious units as in (a).

RNR-S^−^ virus mutants showed obvious attenuation *in vitro*, with poor lytic propagation after BAC DNA transfection into BHK-21 cells. Since plaque assays depend on virus propagation for their readout, we also measured virus titres by flow cytometry of human cytomegalovirus immediate-early 1 promoter-driven viral eGFP expression after overnight infection (eGFP U ml^−1^). As with RNR-L^−^ mutants (Gill *et al.*, 2010), the plaque titres of RNR-S^−^ virus stocks were 10-fold lower than their eGFP titres, whereas for RNR-S^+^ viruses these titres were comparable (data not shown). Therefore to determine the effect of RNR-S disruption on *in vitro* virus growth, we used eGFP^+^ viruses and calculated inputs and measured outputs by eGFP units. RNR-S^−^ mutants showed a moderate replication deficit (Fig. 3a), comparable to that of an RNR-L^−^ mutant (Fig. 3b). The variation in RNR-S deficit between Fig. 3(a) and Fig 3(b) reflects that MuHV-4 lytic replication is far from uniform and can be highly dependent upon parameters such as cell density. Thus comparisons are only ever made within experiments, using viruses titrated at the same time.

### RNR-S^−^ virus luciferase expression *in vivo*

**Fig. 4.**
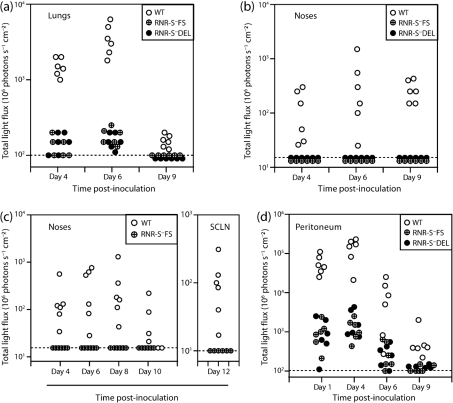
Host colonization by RNR-S^−^ MuHV-4, monitored by luciferase imaging. (a) Mice were inoculated i.n. with WT or RNR-S- (FS, DEL) viruses (10^3^ p.f.u.) in 30 µl under anaesthesia. Infection in different sites was then imaged by injection with luciferin and CCD camera scanning. Each point shows the result for one mouse. WT signals were markedly higher than mutant virus signals, but no mice remained luciferase-negative. (b) Only WT-infected mice gave positive signals in the noses of the same mice. (c) Mice were inoculated i.n. with WT or RNR-S^−^FS viruses (10^3^ p.f.u.) in 5 µl without anaesthesia, then imaged for viral luciferase expression as in (a). No luciferase signals were detected in the lungs. Only WT MuHV-4 gave detectable signals in noses, or at day 12 post-inoculation in the SCLN. (d) Mice were inoculated i.p. with WT or RNR-S^−^ (FS, DEL) viruses (10^3^ p.f.u.), then monitored for viral luciferase expression as in (a). All mice showed positive signals.

We monitored host colonization first by luciferase expression (Fig. 4), since the readout per cell by this method is relatively independent of viral late gene expression (Milho *et al.*, 2009). After intranasal (i.n.) virus inoculation under anaesthesia (Fig. 4a, b), WT luciferase signals were visible in noses and lungs. RNR-S^−^ luciferase signals were also visible in lungs, albeit weaker than those of the WT (Fig. 4a), and were not visible in noses (Fig. 4b). After i.n. virus inoculation without anaesthesia (Fig. 4c), WT luciferase signals were visible in noses, and after 12 days in the draining superficial cervical lymph nodes (SCLN), whereas RNR-S^−^ signals were absent from both sites. After intraperitoneal (i.p.) virus inoculation, both RNR-S^+^ and RNR-S^−^ peritoneal luciferase signals were detected. Again the RNR-S^+^ signal was greater, consistent with RNR-S^−^ mutants being attenuated for *in vivo* lytic spread, but infection was clearly established (Fig. 4d). Thus, it appeared that RNR-S^−^ mutants could infect mice via the lung or peritoneum but not via the nose.

### Establishment and persistence of RNR-S^−^ viral genomes in lymphoid tissue

**Fig. 5.**
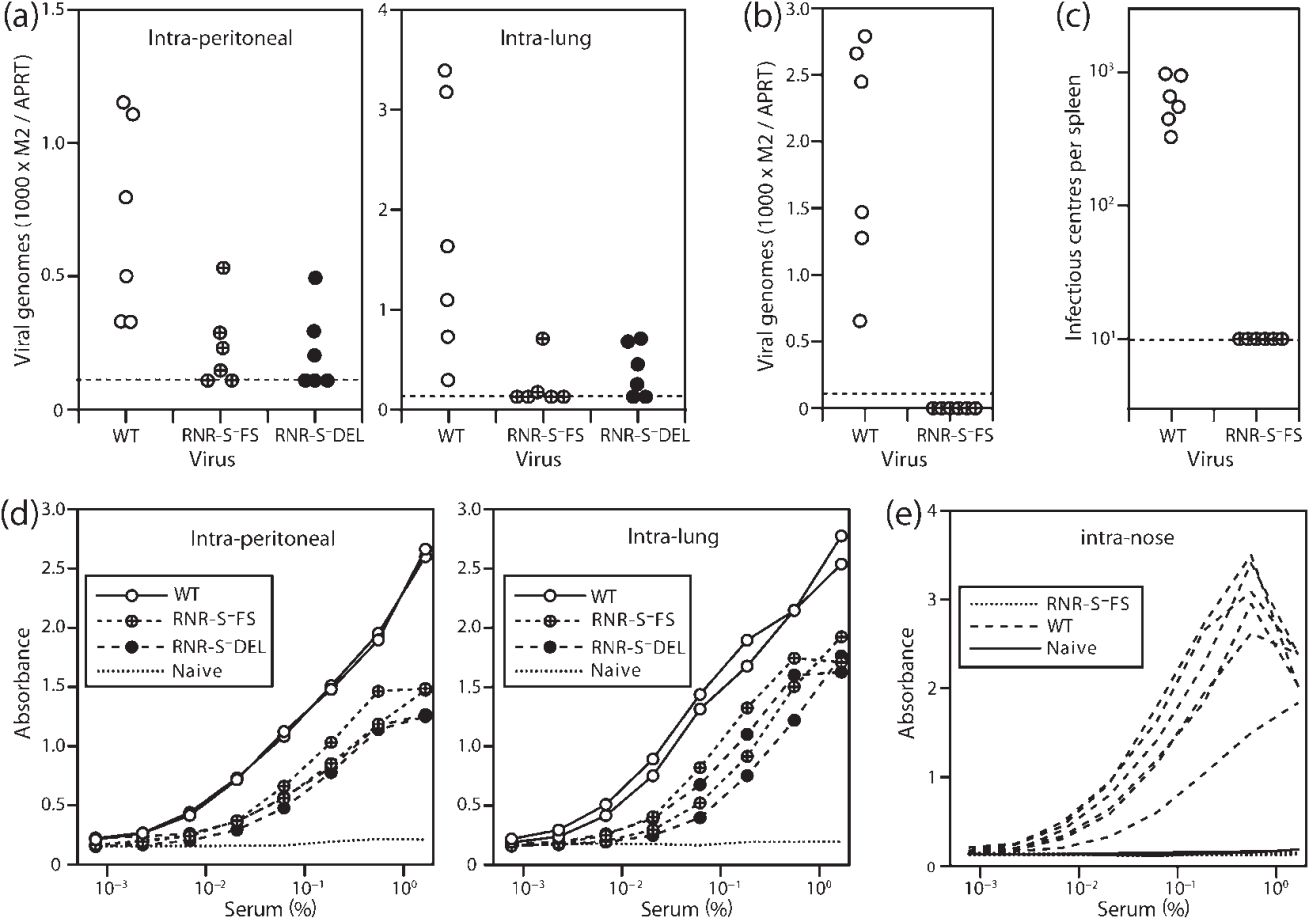
Host colonization by RNR-S^−^ MuHV-4, monitored by PCR for viral DNA and ELISA for virus-specific serum IgG. (a) Mice were infected i.p. or i.n. under anaesthesia (10^3^ p.f.u.) and 3 months later analysed for viral genomes by quantitative PCR of splenic DNA. Viral (M2) copy numbers were normalized by the cellular APRT copy number, amplified in parallel for each sample. Each point shows the result for one mouse. The dashed lines show the detection limit ratio of 0.17, equivalent to five viral copies per reaction. (b). The same analysis was applied to mice 2 months after MuHV-4 inoculation (10^3^ p.f.u.) into just the upper respiratory tract. (c) The mice in (b) were analysed for recoverable virus by infectious centre assay of splenocytes. Each point shows the result for one mouse. The dashed line shows the limit of assay detection. (d) Sera from the mice in (a) were analysed for MuHV-4-specific IgG by ELISA. All showed substantial responses compared with the naive control. Each line shows the mean absorbance reading for three mice. (e) Sera from the mice in (b) were analysed for MuHV-4-specific IgG by ELISA. No antibody response to RNR-S^−^ MuHV-4 was detected. Each line shows the result for one mouse.

Viral luciferase expression depends on a lytic cycle promoter (Milho *et al.*, 2009) that is likely to be shut down during latency. Therefore, we assayed long-term infection by quantitative PCR of viral DNA from spleens 3 months after exposure to RNR-S^+^ or RNR-S^−^ viruses (Fig. 5a, b). RNR-S^−^ genomes were detected in the spleens of 7/12 mice after i.p. inoculation and 6/12 mice after intra-lung inoculation (Fig. 5a). This assay has limited sensitivity as it samples only a small fraction of the total host DNA, and even WT genomes persist at low abundance. Therefore even though RNR-S^−^ viral genomes were not always detected, it seemed that after invasive inoculation RNR-S was not required to establish a persistent infection. In contrast, RNR-S^−^ genomes were not detected in the spleens of any mice 2 months after virus inoculation into the nose (Fig. 5b). Nor were splenic infectious centres (Fig. 5c). Therefore, MuHV-4 required RNR-S to reach the spleen via the upper respiratory tract.

### Antibody response to RNR-S^−^ virus inoculation

To allow for the inherent difficulty of excluding host colonization by a virus that spreads poorly, we also measured MuHV-4-specific serum IgG (Fig. 5d, e). While a positive antibody response would not establish virus persistence, a negative response would argue strongly against a significant infection having occurred. Both i.p. and i.n. (lung) RNR-S^−^ virus inoculations elicited readily detectable antibody responses (Fig. 5d). Thus, ELISA identified evidence of RNR-S^−^ infection having occurred even when PCR of splenic DNA (Fig. 5a) did not. In contrast, only RNR-S^+^ MuHV-4 elicited a detectable antibody response after inoculation into the nose (Fig. 5e). Therefore, RNR-S^−^ MuHV-4 either failed to infect by this route or remained at a very low level.

### Infection by luc^−^RNR-S^−^ MuHV-4

**Fig. 6.**
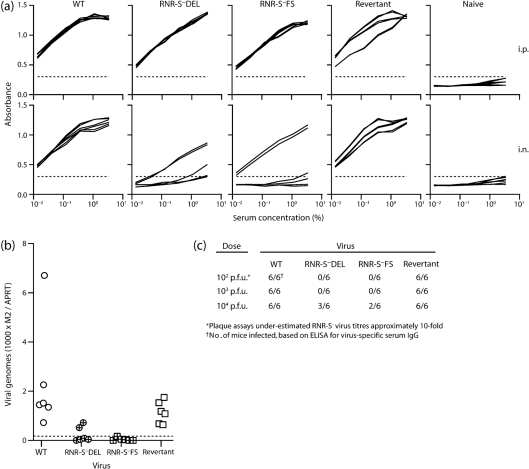
Dose-dependent infection by RNR-S^−^ MuHV-4. (a) Mice were infected (10^4^ p.f.u.) either i.p. or in the upper respiratory tract (i.n.). Sera were tested 1 month later for MuHV-4-specific IgG. Each line shows the result for 1 of 6 mice per group. The dashed line shows the lower detection limit, set by the age-matched naive sera. (b) The mice in (a) were analysed for viral genome carriage by PCR of splenic DNA. Each point shows the result for one mouse, with the viral copy number (M2) normalized according to the cellular DNA copy number (APRT) of the same sample. The dashed line shows the detection limit. (c) The results of ELISAs for MuHV-4-specific serum IgG at 1 month post-inoculation are summarized for different inoculation doses of RNR-S^+^ and RNR-S^−^ viruses. The 10^4^ p.f.u. result is that illustrated in (a).

We also tested infection by luciferase^−^ RNR-S^−^ mutants, using as readouts PCR of viral DNA and ELISA of virus-specific serum IgG (Fig. 6). Mice were infected (10^4^ p.f.u.) either i.p. or in the upper respiratory tract and analysed 1 month later. As with the luciferase^+^ infections, both viral genomes and virus-specific antibodies were detected after i.p. inoculation. However, 3/12 mice given i.n. RNR-S^−^ virus also showed evidence of infection by PCR, and 5/12 did so by IgG ELISA. The fact that mice were infected here, but not by luc^+^RNR-S^−^ MuHV-4 (Fig. 5), most likely reflected the difference in virus dose (10^4^ p.f.u. inoculum versus 10^3^ p.f.u.). We have observed a similar dose dependence with i.n. TK^−^ infection (Gill *et al.*, 2009). With lower doses of luc^−^RNR-S^−^ viruses, ELISAs showed no virus-specific serum IgG (Fig. 6c).

### Histological identification of RNR-S^−^ infection in the nose

**Fig. 7.**
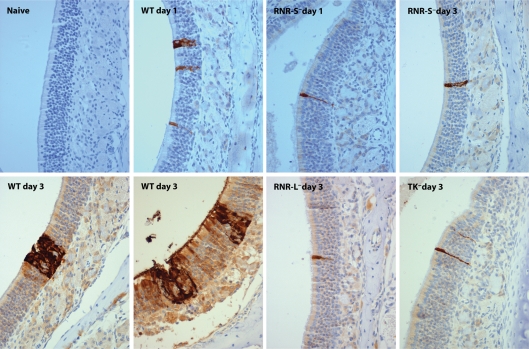
Neuroepithelial infection by MuHV-4 lacking nucleotide metabolism enzymes. Mice were infected i.n. (10^5^ p.f.u., 5 µl, no anaesthesia) with eGFP^+^ MuHV-4. At 1 or 3 days post-inoculation, noses were removed post-mortem, fixed and decalcified. Sections (7 µm) were then stained for viral eGFP expression (brown), and counterstained with Mayer's haemalum (blue). Each section shows a representative area of olfactory neuroepithelium from one mouse. Three mice were analysed at each time point and gave similar results. At 1 day post-inoculation, all viruses were limited to isolated neuroepithelial cells. By day 3 post-inoculation, WT infection had spread, whereas RNR-S^−^, RNR-L^−^ and TK^−^ mutants had not.

To understand better the fate of RNR-S^−^ viruses delivered to the nose, we examined this site by immunohistochemistry (Fig. 7). To avoid the problem of RNR-S deficiency limiting expression of the viral late gene products that are the predominant targets for immune sera, we used eGFP^+^ viruses and identified infected cells at 1 and 3 days post-inoculation by staining for eGFP. At 1 day post-inoculation, WT infection was restricted to isolated cells of the olfactory neuroepithelium. A similar pattern was observed with the RNR-S^−^FS mutant. At 3 days post-inoculation, RNR-S^−^FS infection was still restricted to isolated cells. In contrast, the WT had spread to form clusters of infected neuroepithelial cells. TK^−^ and RNR-L^−^ mutants appeared similar to the RNR-S^−^ mutant. Therefore MuHV-4 could infect the olfactory neuroepithelium without RNR-S, RNR-L or TK, but failed to spread. This explained the lack of luciferase signals in noses, since WT infection is rarely detectable in the nose by CCD camera scanning at 1 day post-inoculation – some virus spread is required to exceed the detection threshold. A lack of late viral lytic gene expression or spread similarly explained the lack of a detectable antibody response to RNR-S^−^ virus in the upper respiratory tract.

We occasionally observed infection of the nasal respiratory epithelium. This was much less common than neuroepithelial infection. It possibly represented a low efficiency infection route to explain the occasional host colonization achieved by high dose RNR-S^−^ virus inoculations (Fig. 6).

## Discussion

Epidemiology can reveal likely modes of gammaherpesvirus transmission, but rarely the primary cells that incoming virions target or how infection then spreads. This is important, because gammaherpesviruses may infect different cell types in different ways (Hutt-Fletcher, 2007; Shannon-Lowe *et al.*, 2009), mandating different interventions for infection control. Here, efficient host entry via the upper respiratory tract required viral enzymes that function in nucleotide supply. Each enzyme may also have evolved new functions. However, the similar phenotypes of three separate gene disruptions (TK, RNR-L and now RNR-S) would argue that their common function of nucleotide supply is what host entry requires. Histological analysis provided a plausible explanation: MuHV-4 must infect and replicate lytically in differentiated olfactory neuroepithelial cells in order to reach the B-cells that provide its normal latent reservoir.

The different requirements for host entry between nose, lung and peritoneal cavity argued that inoculation route is an important consideration when extrapolating experimental results to a more natural setting. Aerosolized, virus-laden droplets can in theory reach the lung without anaesthesia, but the distance to travel and possibilities for capture en route are very great, implying that this would require copious virus shedding, and there is no epidemiological evidence for gammaherpesvirus transmission in this way. Therefore, inoculating MuHV-4 into lungs must be considered artificially invasive. MuHV-4 (10^4^ p.f.u. of WT) delivered i.n. in 5 µl never infects the lungs if given without anaesthesia (Milho *et al.*, 2009), and rarely infects the lungs even if given under general anaesthesia (data not shown). A rough scaling factor of 3000 would make 5 µl in a mouse equivalent to 15 ml in a human – a considerably greater volume than is likely to be involved in natural transmission. Upper respiratory tract infection, by contrast, works even with a 1 µl inoculum. Thus while MuHV-4 DNA can persist without lytic replication if it reaches a suitable site (Tibbetts *et al.*, 2006; Moser *et al.*, 2006; Kayhan *et al.*, 2007), unless the normal barriers to host entry are bypassed RNR and TK are required to reach such a site. MuHV-4 may survive for some time in the olfactory neuroepithelium, but seems not to colonize the nervous system after intranasal inoculation (Terry *et al.*, 2000), and so is unlikely to persist in neurons or glial cells. We have found it difficult to recover even WT MuHV-4 from the olfactory neuroepithelium in the long term.

An olfactory entry route makes sense for a murid herpesvirus, because it exploits normal host environmental sampling to achieve efficient virion uptake. Human noses, being further from the ground, might seem less suitable targets. However, gammaherpesvirus–host relationships were established long before humans evolved. Therefore the general scheme of host entry proceeding via terminally differentiated epithelial cells and so requiring viral nucleotide metabolism enzymes seems likely to be conserved, particularly between rhadinoviruses. (MuHV-4 and KSHV are more closely related to each-other than either is to EBV.) Although the main role defined for HSV nucleotide metabolism enzymes is to reactivate from neurons rather than to infect them in the first place, experimental HSV delivery typically involves scarification; a less invasive inoculation might give different results. We find that TK^+^ but not TK^−^ vaccinia virus elicits an antibody response after upper respiratory tract inoculation, whereas both elicit antibody responses after i.p. inoculation (data not shown). Therefore many DNA viruses may encounter terminally differentiated cells when they first enter new hosts, and so need to boost cellular deoxyribonucleotide production in order to spread.

A requirement for viral lytic replication to reach a viable latent reservoir opens up the possibility of arresting gammaherpesvirus infections at a very early stage with nucleoside analogues. However, such treatments would have to be based on a suspicion of contact rather than symptoms. Thus they would probably be restricted to settings such as immunocompromised patients inadvertently exposed to primary infection. Also we do not know how long the initial neuroepithelial infection remains viable for further dissemination. Therefore considerable hurdles remain. Nevertheless, the requirement for multiple viral genes – RNR-S, RNR-L, TK and presumably also the viral DNA polymerase – indicates that such treatments would have a range of possible targets.

## Methods

### 

#### Mice.

Female BALB/c mice (6–12 weeks old) were infected i.n. with MuHV-4 (10^3^ p.f.u. unless stated otherwise), either in 30 µl under general anaesthesia to infect both upper and lower respiratory tracts, or in 5 µl without anaesthesia to infect just the upper respiratory tract. I.p. infections were with 10^3^ p.f.u. in 300 µl. To monitor viral luciferase expression, mice were injected i.p. with luciferin (2 mg per mouse) and scanned with an IVIS Lumina CCD camera (Caliper Life Sciences) (Milho *et al.*, 2009). Quantitative comparisons used the maximum radiance (photons s^−1^ cm^−2^) over each region of interest. All experiments conformed to local animal ethics regulations and to Home Office Project Licence 80/1992.

#### Plasmids and viruses.

The MuHV-4 RNR-S is encoded by ORF60 (genomic co-ordinates 80479–79562), and RNR-L by ORF61 (genomic co-ordinates 82865–80514) (Virgin *et al.*, 1997). Fluorescent tagged RNR subunits were cloned by PCR amplification (Phusion DNA polymerase; New England Biolabs) of ORF60 with *Eco*RI/*Bam*HI restricted primers and of ORF61 with *Xho*I/*Bam*HI restricted primers. ORF61 was cloned into the *Xho*I/*Bam*HI sites of pEGFPC2 (Clontech), thereby adding eGFP to its N terminus. ORF60 was cloned into the *Eco*RI/*Bam*HI sites of a pEGFPC2 derivative in which the mCherry coding sequence replaces that of eGFP, thereby fusing mCherry to the ORF60 N terminus.

An RNR-L mutant has been described previously (Gill *et al.*, 2010). To make an ORF60-disrupted virus, we PCR-amplified genomic co-ordinates 81534–80265, including *Bam*HI and *Sac*I restriction sites in the primers (5′-GAGGGATCCATTGAGAGGCTGGAGAGGG-3′; 5′-CAGGAGCTCGATGTTAAAGTTGACCAGCCTC-3′), and cloned the PCR product into the same sites of pSP73 (Promega Corporation). We then amplified two alternative second flanks, from the *Bgl*II restriction site at genomic co-ordinate 78718 (5′-CATACAATCAAAGATCTAATCAAAC-3′) to either genomic co-ordinate 79793 (5′-GGAGAGCTCCTTTATTGCTGCCAAGAGCAAGA-3′) or 80261 (5′-TCGGAGCTCTGGTCAAGGAGTTTAATTGCCATG-3′). A *Sac*I restriction site was included in each second primer, and the PCR product was cloned into the *Sac*I/*Bgl*II sites of pSP73. Thus the two genomic flanks, separated by a *Sac*I site, incorporated either a frameshift in the RNR-S coding sequence after amino acid residue 72 (RNR-S^−^FS), or a deletion of genomic co-ordinates 80264–79794 (RNR-S^−^DEL). Each construct was subcloned as a *Bgl*II–*Bam*HI fragment into the *Bam*HI site of the pST76K-SR shuttle vector, and then recombined into a MuHV-4 BAC by transient RecA expression (Adler *et al.*, 2000). A revertant of the RNR-S-FS mutant BAC was made by reconstituting the corresponding unmutated genomic fragment. We also generated luciferase^+^ RNR-S-FS and RNR-S-DEL mutants by shuttling the same ORF60 disruptions into an MuHV-4 BAC in which luciferase is expressed from an ectopic viral lytic promoter in the ORF57–ORF58 intergenic site (Milho *et al.*, 2009). Recombinant BACs were tested for genomic integrity by restriction endonuclease mapping and by DNA sequencing across the mutation site. Recombinant viruses were recovered by transfecting BAC DNA into BHK-21 cells. For *in vivo* experiments, the loxP-flanked viral BAC–eGFP cassette was removed by passage through NIH-3T3-CRE cells (Stevenson *et al.*, 2002). Virus stocks were grown in BHK-21 cells and recovered from infected cell supernatants by ultracentrifugation (de Lima *et al.*, 2004).

#### Cells and mAbs.

BHK-21 cells, HeLa cells and NIH-3T3-CRE cells were cultured in Dulbecco's modified Eagle's medium (DMEM; Invitrogen Corporation) supplemented with 2 mM glutamine, 100 U penicillin ml^−1^, 100 mg streptomycin ml^−1^ and 10 % FCS (PAA Laboratories). Cells were transfected using either Fugene-6 (for BAC DNA) or Lipofectamine (for expression plasmids). B-cell hybridomas were generated by fusing splenocytes with NS0 cells. Hybrids were cultured on irradiated MRC-5 feeder cells in RPMI 1640 supplemented as for DMEM, and selected with 1 mg azaserine ml^−1^ plus 100 mM hypoxanthine (Gillet *et al.*, 2007). We isolated two IgG_2a_ mAbs specific for RNR-L (PS-8A7 and BZ-5B2) and one IgG_2a_ mAb specific for RNR-S (PS-10A10).

#### Virus assays.

Virus stocks were titrated by plaque assay on BHK-21 cells (de Lima *et al.*, 2004). Cell monolayers were incubated with virus dilutions (2 h, 37 °C), overlaid with 0.3 % carboxy-methylcellulose, and 4 days later fixed and stained for plaque counting. Latent virus in spleens was measured by infectious centre assay (de Lima *et al.*, 2004). Viral genome loads were measured by real-time PCR (Gaspar *et al.*, 2008). DNA (50 ng) was extracted from *ex vivo* organs (Wizard genomic DNA purification kit; Promega Corporation) and used to amplify MuHV-4 genomic co-ordinates 4166–4252 (M2 gene) (Rotor Gene 3000; Corbett Research). The PCR products were quantified by hybridization with a Taqman probe (genomic co-ordinates 4218–4189) and converted to genome copies by comparison with a standard curve of cloned plasmid template amplified in parallel. Cellular DNA was quantified in the same reaction by amplifying part of the adenosine phosphoribosyltransferase (APRT) gene (forward primer 5′-GGGGCAAAACCAAAAAAGGA-3′, reverse primer 5′-TGTGTGTGGGGCCTGAGTC-3′, probe 5′-TGCCTAAACACAAGCATCCCTACCTCAA-3′), again with plasmid template dilutions amplified in parallel for quantification. Virus loads were then expressed relative to the cellular genome copy number of each sample.

#### ELISA.

MuHV-4 virions recovered from infected cell supernatants were disrupted with 0.05 % Triton X-100 in 50 mM sodium carbonate (pH 8.5), and coated (18 h, 4 °C) onto Maxisorp ELISA plates (Nalge Nunc). The plates were washed three times in PBS/0.1 % Tween-20, blocked with 2 % BSA in PBS/0.1 % Tween-20 (1 h, 23 °C), and incubated with threefold serum dilutions (1 h, 23 °C). The plates were then washed four times in PBS/0.1 % Tween-20, incubated (1 h, 23 °C) with alkaline phosphatase-conjugated goat anti-mouse IgG-Fc pAb (Sigma Chemical Co.), washed five times, and developed with nitrophenylphosphate. Absorbance was read at 405 nm with a Bio-Rad Benchmark ELISA plate reader.

#### Southern blotting.

Virions were recovered from infected cell supernatants by ultracentrifugation. DNA was extracted from them by alkaline lysis, digested with *Eco*RI, electrophoresed on a 0.8 % agarose gel and transferred to positively charged nylon membranes (Roche Diagnostics Ltd). A ^32^P-dCTP-labelled probe (APBiotech) was generated by random primer extension (Nonaprimer kit; Qbiogene) of a cloned ORF60 template. Membranes were hybridized with the probe (65 °C, 18 h), washed to a stringency of 0.2 % SSC, 0.1 % SDS and exposed to X-ray film.

#### Immunofluorescence.

MuHV-4-infected BHK-21 cells or transfected HeLa cells were fixed in 4 % paraformaldehyde (30 min), then washed in PBS and permeabilized with 0.1 % Triton X-100. Viral eGFP expression was visualized directly. Other viral proteins were detected with MuHV-4-specific mAbs plus Alexa 568-coupled goat anti-mouse IgG pAb (Invitrogen Corporation), washing the cells three times with PBS/0.1 % Tween-20 after each antibody incubation. Nuclei were counterstained with DAPI. Fluorescence was visualized by using a Leica confocal microscope or an Olympus fluorescence microscope with digital image capture (Q Imaging).

#### Flow cytometry.

Cells exposed to eGFP^+^ viruses were trypsinized and analysed for green channel fluorescence on a FACSort using Cellquest (BD Biosciences).

#### Immunohistochemistry.

The anterior part of the skull containing the olfactory epithelium was removed post-mortem and fixed in 4 % formaldehyde–PBS (4 °C, 24 h). Samples were decalcified by gentle agitation in 150 mM NaCl, 50 mM Tris/HCl (pH 7.2), 270 mM EDTA (2 weeks, 23 °C), changing the buffer every 3 days, then washed twice in PBS and paraffin-embedded. Sections (7 µm) were de-paraffinized in xylene and rehydrated through graded ethanol solutions. Antigen retrieval was performed by microwaving two times for 5 min in 10 mM sodium citrate (pH 6), 0.05 % Tween-20. Endogenous peroxidase activity was quenched in PBS with 3 % H_2_O_2_ for 10 min. Sections were blocked with the Avidin/Biotin Blocking kit (Vector Laboratories) and in PBS with 2 % goat serum, 2 % BSA (1 h, 23 °C). Infected cells were detected with rabbit anti-eGFP pAb (Abcam) (18 h, 23 °C). Subsequently, sections were incubated for 30 min in biotinylated goat anti-rabbit IgG pAb (Vector) and the Vectastain Elite ABC Peroxidase system with ImmPACT DAB substrate (Vector Laboratories). Sections were washed in PBS between each step. Finally, the sections were counterstained with Mayer's Hemalum (Merck), dehydrated and mounted in DPX (BDH Chemicals).
